# Electrical and Electrochemical Properties of Conducting Polymers

**DOI:** 10.3390/polym9040150

**Published:** 2017-04-23

**Authors:** Thanh-Hai Le, Yukyung Kim, Hyeonseok Yoon

**Affiliations:** 1 Department of Polymer Engineering, Graduate School, Chonnam National University, 77 Yongbong-ro, Buk-gu, Gwangju 61186, Korea; hanhhai1989@gmail.com (T.-H.L.); ykkim6025@gmail.com (Y.K.); 2 School of Polymer Science and Engineering, Chonnam National University, 77 Yongbong-ro, Buk-gu, Gwangju 61186, Korea

**Keywords:** conducting polymers, conductivity, electronic properties, electrochemistry, pseudocapacitors, sensors

## Abstract

Conducting polymers (CPs) have received much attention in both fundamental and practical studies because they have electrical and electrochemical properties similar to those of both traditional semiconductors and metals. CPs possess excellent characteristics such as mild synthesis and processing conditions, chemical and structural diversity, tunable conductivity, and structural flexibility. Advances in nanotechnology have allowed the fabrication of versatile CP nanomaterials with improved performance for various applications including electronics, optoelectronics, sensors, and energy devices. The aim of this review is to explore the conductivity mechanisms and electrical and electrochemical properties of CPs and to discuss the factors that significantly affect these properties. The size and morphology of the materials are also discussed as key parameters that affect their major properties. Finally, the latest trends in research on electrochemical capacitors and sensors are introduced through an in-depth discussion of the most remarkable studies reported since 2003.

## 1. Introduction

Over the past several decades, conducting polymers (CPs) have gained increasing attention owing to their strong potential as alternatives to their inorganic counterparts, leading to significant fundamental and practical research efforts. In the late 1970s, many scientists considered CPs (or ‘synthetic metals’) to be intractable and insoluble. Since the discovery of polyacetylene in 1977 by Hideki Shirakawa, Alan MacDiarmid, and Alan Heeger, various important CPs have been investigated continuously, including polypyrrole (PPy), polyaniline (PANI), polythiophene (PT), poly(3,4-ethylenedioxythiophene) (PEDOT), trans-polyacetylene, and poly(*p*-phenylene vinylene) (PPV) [1]. In general, CPs possess alternating single (σ) and double (π) bonds, and these π-conjugated systems lend the CPs their inherent optical, electrochemical, and electrical/electronic properties. It is known that the parameters that most affect the physical properties of CPs are their conjugation length, degree of crystallinity, and intra- and inter-chain interactions.

CPs provide the advantages of chemical diversity, low density, flexibility, corrosion resistance, easy-to-control shape and morphology, and tunable conductivity over their existing inorganic counterparts [2,3]. However, the development of the properties of CPs has not been completely commensurate with those of their metallic and inorganic semiconductor counterparts. Consequently, CPs have been modified or hybridized with other heterogeneous material components to overcome their inherent limitations in terms of solubility, conductivity, and long-term stability. Judicious coupling of CPs with other materials can result in materials with attractive properties and new application opportunities in diverse fields ranging from electronics to energy devices. Researchers in this field have reported a variety of strategies to obtain CP-based composites and hybrids with novel structures and improved properties. As a typical example, CP nanocomposites containing carbon nano-species such as graphene, carbon nanofibers, and carbon nanotubes have been developed [4,5,6,7]. These carbon nano-species improved the structural ordering of the CP chains and facilitated delocalization of the charge carriers, resulting in enhanced conductivity. A rich spectrum of conductivities has been achieved, ranging from insulating to metallic [2,3,8].

Successful preparations of CP composites with high mechanical stabilities, flexibilities, and conductivities have proven that CPs can serve as key material components in light emitting diodes [9,10], transistors [11,12], electrochromic devices [13,14], actuators [15,16], electrochemical capacitors [17,18], photovoltaic cells [19,20], and sensors [21,22]. The most crucial factor for progress in such fields is achieving control of the electrical or electrochemical properties of CPs. Consequently, this review presents a discussion of the electrical and electrochemical properties of CPs, and the latest trends in research on the applications of CPs have been summarized.

## 2. Conductive Mechanism

### 2.1. Inherent Molecular Structure

The electrical conductivity of a material is mainly determined by its electronic structure. The energy band theory is a useful way to visualize the differences among conductors, insulators, and semiconductors. The band gap is the energy difference between the valence and conduction bands of a material. When the valence band overlaps the conduction band, the valence electrons are free to move and propagate in the conduction band. This is an intrinsic characteristic of conductors. Semiconductors possess small energy gaps that electrons can cross upon excitation to reach the conduction band, leaving behind a hole. This allows both hole and electron charge transport, which allows the conduction of current. In the case of insulators, the band gap is too large to be crossed by electrons, and therefore they do not conduct electricity.

**Figure 1:**
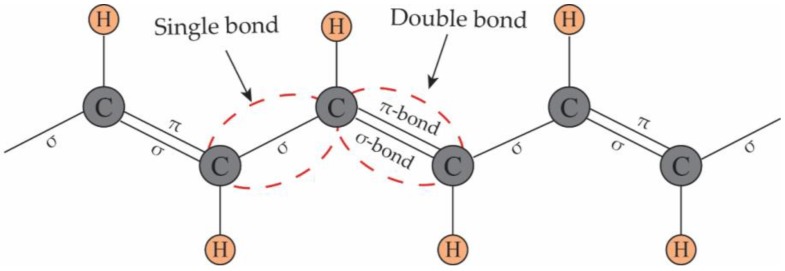
The structure of polyacetylene: The backbone contains conjugated double bonds.

However, the energy band theory does not clearly explain why CPs, being organic materials, conduct electricity. Many studies have addressed the transport properties of CPs at the molecular level [23,24]. Here, polyacetylene is used as an example to illustrate the principles of conduction in CPs (Figure 1) because of its simple chemical structure and remarkably high electrical conductivity. From the perspective of chemists, the common electronic feature of pristine CPs is the presence of conjugated single and double bonds along the polymer skeleton. Both single and double bonds include a localized σ-bond, which forms a strong chemical bond. Additionally, each double bond also contains a localized π-bond, which is weaker [25,26].

The π-bond between the first and second carbon atoms is transferred to the position between the second and third carbon atoms. In turn, the π-bond between the third and fourth carbon travels to the next carbon, and so on. As a result, the electrons in the double bonds move along the carbon chain (The p_z_-orbitals in the chain of π-bonds overlap continuously and the electrons in the π-bonds thus move along the carbon skeleton). Thus, conjugated double bonds allow electric flow. However, conjugated bonds do not render polymeric materials highly conductive. A breakthrough by Shirakawa, Heeger, and MacDiarmid was achieved by exploiting the complementary ways in which chemists and physicists think about conductance. Their work concerned a system in which a halogen dopant removes an electron from a delocalized bonding arrangement creating a hole. Then, an electron at a neighboring position jumps and fills that hole, generating a new hole and allowing charge to flow through the polymer chain. Since the publication of this pioneering work, many theories regarding the conductivity of CPs have been developed, the overwhelming majority of which attribute changes in the conductivity of CPs to the formation of nonlinear local excitations (e.g., solitons, polarons, and bipolarons) as charge carriers [24,27,28].

### 2.2. Doping

CPs have been doped using different methods in order to achieve high conductivities [29]. Un-doped polymers have been reported as insulators but, upon doping, their conductivity can change from insulating to metallic. Owing to their unique chemical structures, however, the doping mechanism for CPs is completely different to that for their inorganic counterparts. Dopants in the polymer undergo redox processes in which charges are transferred with subsequent formation of charge carriers [30]. The role of the dopant is not only to withdraw electrons from the CP but also to add electrons to the CP backbone. A simple explanation of the effect of doping is that electrons are extracted from the highest occupied molecular orbital (HOMO) of the valence band (oxidation) or transferred to the lowest unoccupied molecular orbital (LUMO) of the conduction band (reduction). This oxidation/reduction process creates charge carriers in the form of polarons (radical ions), bipolarons (dications or dianions), or solitons in the polymer. CPs can be categorized into degenerate and non-degenerate systems based on their bond structures in the ground state. Degenerate polymers possess two identical geometric structures in the ground state while non-degenerate polymers exhibit two different structures with different energies in the ground state (e.g., benzenoid and quinoid structures, where the energy of the benzenoid is lower than that of the quinoid). Solitons are known to be the charge carriers in degenerate systems such as polyacetylene. Conversely, polarons and bipolarons serve as the charge carriers in both degenerate and non-degenerate systems such as PPy and PT [31,32]. The movement of these charge carriers along polymer chains produces conductivity. In solid-state physics terminology, the oxidation and reduction processes correspond to *p*-type and *n*-type doping, respectively [10]. In *p*-type doping, the electron moves directly from the HOMO of the polymer to the dopant species and creates a hole in the polymer backbone. Conversely, in *n*-type doping, electrons from the dopant species move to the LUMO of the polymer, resulting in increased electron density. Hence, the density and mobility of charge carriers can be tuned by doping [33,34,35,36]. 

**Figure 2:**
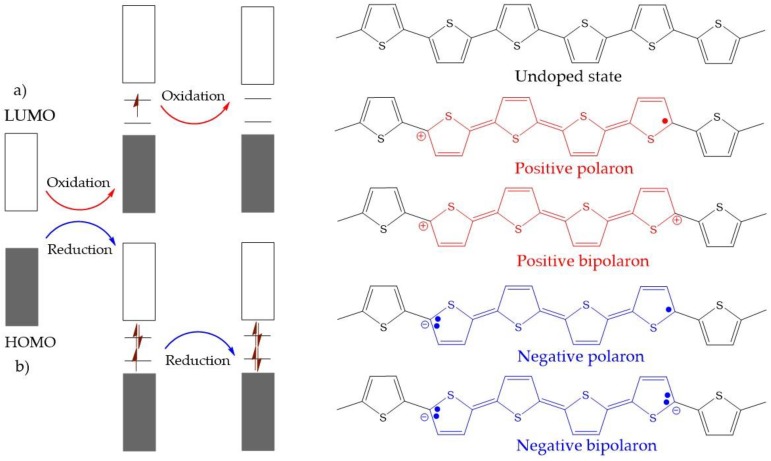
The electronic band and chemical structures of polythiophene (PT) with (**a**) *p*-type doping and (**b**) *n*-type doping.

**Figure 3:**
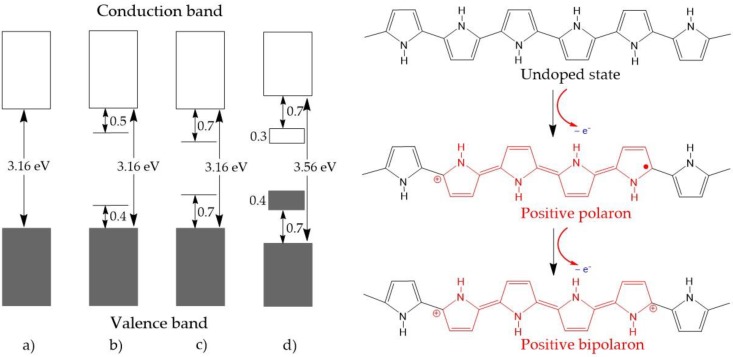
Electronic bands and chemical structures illustrating (**a**) undoped; (**b**) polaron; (**c**) bipolaron; and (**d**) fully doped states of polypyrrole (PPy).

CPs can undergo both *p*-type doping and *n*-type doping, as shown in Figure 2. The doping process generates positive or negative polarons/bipolarons. These charge carriers are delocalized over the polymer chains, which facilitates the electronic conductivity. Generally, the negatively charged carriers in *n*-doping are not as stable as positively charged forms, which makes *p*-doping more popular in academic research as well as for practical applications. Representatively, the conductivity in PPy is an outcome of *p*-type doping. The PPy chain exhibits four distinct electronic band structures with different doping levels. In the undoped state, PPy is an insulator with a large band gap of approximately 3.16 eV (Figure 3a). Upon oxidation, a π-electron is removed from the neutral PPy chain, and a local deformation from the benzenoid structure to a quinoid one occurs to form a polaron [24,37]. This gives rise to two localized electronic levels within the band gap while the unpaired electron occupies the bonding state (Figure 3b). Upon further oxidation, a second electron is removed from the PPy chain, resulting in the formation of a doubly charged bipolaron (Figure 3c). The benzenoid-to-quinoid deformation is stronger in the bipolaron than in the polaron. As the polymer is further oxidized, an overlap between bipolarons occurs, leading to the formation of two narrow bipolaronic bands (Figure 3d) and a decrease in the energy gap from 3.16 to 1.4 eV.

**Figure 4:**
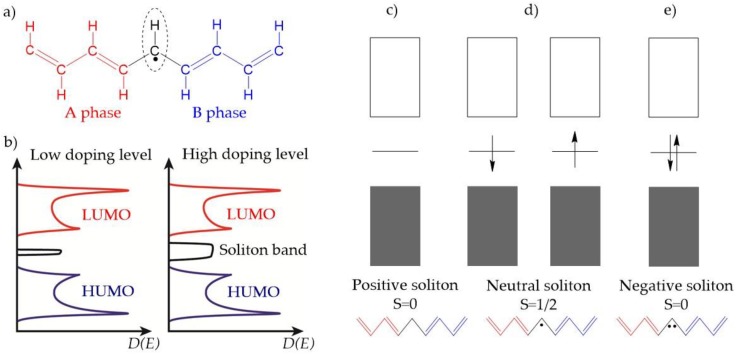
(**a**) Schematic illustration of the geometric structure of a neutral soliton on a trans-polyacetylene chain; (**b**) A soliton band with light doping (left) and heavy doping (right); The band structure of trans-polyacetylene containing (**c**) a positively charged soliton; (**d**) a neutral soliton; and (**e**) a negatively charged soliton.

Trans-polyacetylene is a typical CP with a degenerate ground state. When the chain comprises an odd number of carbons, the single and double bonds can exchange electrons, leading to two geometric structures (A and B phases) with the same energy (Figure 4a). A radical form generated between the two structures contains an unpaired π electron (Figure 4d). This defect has been called a (neutral) soliton. The soliton possesses a certain mobility that allows its delocalization along the polymer backbone. When the neutral solitons move along the chain and meet one another, they can be destroyed by forming a double bond. If the solitons have charge, they become more stable as they can be delocalized over the polymer chain. A neutral soliton can be oxidized or reduced by a dopant to form a positive or negative soliton. Positive and negative solitons possess positive and negative charges with no unpaired spin (S) (Figure 4c,e). In the polymer backbone, the π electron travels over a long distance and the regions of individual charged solitons can overlap. The interaction between charged solitons leads to a band-like feature called the soliton band [38]. The soliton band is located in the middle of the HUMO and LUMO of the polymer and can become larger with an increase in doping level (Figure 4b). Both solitons and polarons act as charge carriers that facilitate electronic conductivity in trans-polyacetylene [24].

There are significant extrinsic, environmental factors that influence the conductivity of CPs, such as temperature, as well as intrinsic ones such as the degree of doping. We will discuss these factors in Section 3.

## 3. Electrical Properties

### 3.1. Tunable Conductivity

In polyconjugated systems, the behavioral properties of the π-electrons, such as their delocalization and polarization, play significant roles in determining the electrical properties of the system. In first-generation CPs the maximum conductivity was limited because of pronounced disorder in the polymer matrix. Structural and morphological disorder inhibits π-electron delocalization, thus retarding charge transport [27,39]. As a result, the metallic charge conduction of first-generation CPs was rather weak.

**Figure 5:**
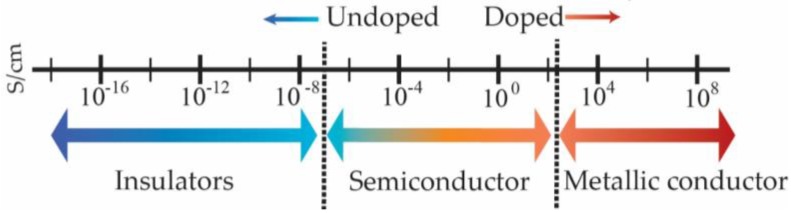
The general conductivity range of conducting polymers (CPs).

As discussed in previous sections, the formation of a metallic state in CPs upon doping has been reported. When π-conjugated systems are doped, their structural and morphological disorder is reduced. This helped to create a new generation of CPs in which the conductivity of the pristine polymer can be raised from the insulating to the metallic regime. Charges generated through doping exist as solitons, polarons, and bipolarons, which can be accompanied by lattice distortion [40]. The conductivity of undoped polymers is 10^−6^–10^−10^ S·cm^−1^, lying at the boundary region between semiconductor and insulator (Figure 5). The conductivity of undoped polymers can be increased by 10 or more orders of magnitude through doping. For instance, Tsukamoto et al. [41,42] reported the doping of polyacetylene with iodine and achieved a conductivity of more than 10^4^ S·cm^−1^, which is comparable with the conductivity of lead at room temperature (4.8 × 10^4^ S·cm^−1^). With the achievement of such high conductivity, CPs became a promising candidate material for electronic applications.

### 3.2. Charge Carrier Transport Models

The charge transport properties of CPs are strongly governed by the disorder stemming from sp^2^ defects in the polymer chain and chain ends, chain entanglement, voids, and doping defects. In 1958, Anderson introduced the concept of localization to describe electronic transport in a homogeneously disordered material. In a perfect crystal with periodic potentials, the wave functions form Bloch waves delocalized throughout the material. However, disorder can alter the wave function. More specifically, in disordered systems, impurities and structural defects introduce substantial scattering of the electronic wave function, which may lead to localization. In the presence of strong disorder, the overlap of the wave functions decreases exponentially and the system moves towards the insulating regime. The localized wave function has the form

(1) $$\Psi \left(r\right)\;\infty \;\exp\left(-\frac{r-r_{0}}{\xi }\right)$$

where *ξ* is the localization length of the state. Mott, years later, pointed out that states at the center of the band are delocalized, while states at the band tail are more easily localized since these states are formed from localized orbitals [43,44]. The term “mobility edge” was coined to refer to the critical energy (*E*_c_) separating the extended and localized states. The mobility edge is associated with the transition between a metal and an insulator. The electronic properties of materials depend on the position of the Fermi level (*E*_F_) and *E*_c_. When *E*_F_ lies in the region of localized states, the material shows nonmetallic behavior, even though there is a finite density of states at the Fermi level. In contrast, when *E*_F_ lies in the region of extended states, the material has a finite DC conductivity (*σ*) and exhibits metallic behavior at low temperature (when the temperature (*T*) tends to zero K). The DC conductivity is found from Drude or Boltzmann theory, as electrical conductivity is mainly determined by

(2) $$\sigma =\frac{ne^{2}\tau }{m}$$

where *n* is the carrier density, $\tau =\frac{l}{v_{F}}$ is the relaxation time, *m* is the effective mass of the carrier, and *e* is the electron charge [27,44,45,46]. However, Equation (2) only applies for weak disorder. Weak disorder means that the mean free path l is much greater than the Fermi wavelength *k*_F_^−1^, i.e., $k_{F}l\gg 1$, in the metallic system.

(3) $$k_{F}l=\frac{\left[h{\left(3\pi^{2}\right)}^{\frac{2}{3}}\right]}{e^{2}\rho n^{1/3}}$$

where *ρ* is the electrical resistivity. The Anderson concept does not clearly explain the dielectric response behavior of highly doped CPs. Epstein et al. [47] proposed an inhomogeneous disorder model involving 3D metallic crystalline domains separated by a disordered quasi-1D medium to explain the delocalization of charges in CPs [48,49]. This model agrees with the frequency-dependent results of studies on PANI and PPy [47,48,49]. Another model, termed ‘variable range hopping’, explains charge transport based on the hopping of charge carriers in disordered systems or charging energy-limited tunneling between domains [50,51]. In fully doped CPs, charge-carrier density is of the order of 10^21^ cm^−3^, l is around 10 Å, and $k_{F}l$ ≈ 1−10 at room temperature. To date, a metallic state has been observed in six different CPs, as listed in Table 1.

The strength of the conductivity is reported to strongly depend on the nature and concentration of the dopant and doping time, suggesting a complex mechanism for the doping process. A great deal of research has attempted to increase the conductivity of CPs by varying the dopant. Based on their molecular size, dopants can be categorized into small cations/anions (e.g., Na^+^, Cl^−^ and ${\mathrm{ClO}}_{4}^{-}$) and large polymeric species (e.g., polystyrene sulfonate and polyvinyl sulfonate). Typical examples of dopants used for CPs and the conductivity values obtained therefrom are listed in Table 2. The nature of the dopants affects not only the conductivity but also the surface and structural properties of CPs. Large dopants can change the polymer density and thus affect the surface topography and physical properties of the polymer. In addition, large dopants can be strongly bound to the polymer, which prevents the leaching of the dopant molecules from the polymer matrix, even under extreme conditions. Small dopants, in contrast, can be readily inserted/de-inserted or exchanged with other ions existing in the surrounding environment [52].

For most CPs, conductivity increases with increasing doping level. An increase in the electrical conductivity resulting from an increase in the dopant concentration was described in detail by Tsukamoto et al. for stretched polyacetylene doped with controlled amounts of I_2_ [41,42]. They observed that the conductivity of the polymer increased stepwise owing to the formation of ordered stacking structures. The conductivity became saturated after several hours of doping. This extended doping time indicated the slow diffusion of the dopant ions into the polymer, suggesting that the polymer had dense, ordered structures [41,42]. The electrical conductivity of CPs increases with the dopant concentration and becomes saturated at high doping levels. The doping/de-doping process is reversible, i.e., de-doping usually reproduces the original, undoped CP without degradation of the polymer backbone.

A number of methods have been explored for doping CPs. The available methods are summarized in Table 3, and include electrochemical doping, chemical doping, photodoping, non-redox doping, and charge-injection doping [53,54,55]. The first two techniques are widely used because of their low cost and convenience. Chemical doping may take the form of vapor-phase doping and solution doping. In vapor-phase doping, polymers are exposed to the vapors of dopant compounds such as iodine, bromine, AsF_5_, and SbF_5_. The level of doping is determined by the vapor pressure and reaction time. Solution doping uses a solvent in which the dopant and the products formed during doping are soluble. Electrochemical doping is accomplished by applying a DC power source between a CP-coated positive electrode and a negative electrode. This electrochemical approach offers precise control of the doping level by monitoring the current passed. In this system, the electrode supplies the redox charge to the polymer, and ions diffuse from the electrolyte into the polymer to compensate the charge. Compared with chemical approaches, electrochemical doping is easy to control and more readily reversible.

Over time, improvements in the processing of these CPs have led to greater conductivity being achieved, and both *n*-type and *p*-type dopants have been used to enhance the electrical conductivity of CPs.

### 3.3. Temperature Dependence

The conductivities of fully doped polymers are comparable to those of conventional metals. The critical temperature dependence of several CPs, including polyacetylene, PPy, PPV, and PANI, has been investigated. For all of these polymers, the characteristic temperature dependence of the electronic conductivity has been explained by dividing these materials into three regimes according to their reduced activation energy (*W*), identified using Zabrodski plots:

(4) $$W\left(T\right)=-\frac{T\left[\mathrm{dln}\rho \left(T\right)\right]}{dT}=\frac{d\left(\ln\sigma \right)}{d\left(\ln T\right)}$$

In the insulating regime, the resistivity is activated and σ follows a Mott variable range hopping mode:

(5) $$\sigma =\sigma_{0}\exp{\left(\frac{T_{0}}{T}\right)}^{1/\left(n+1\right)}$$

*W*(*T*) has a negative temperature coefficient and can be determined as [77]

(6) $$\log_{10}W\left(T\right)=A-x\log_{10}T$$

where $A=x\log_{10}T_{0}-\log_{10}x$ [77]. From a plot of $\log_{10}W$ against $\log_{10}T$ (as represented in Equation (6)), the slope *x* and dimensionality of the sample can be obtained.

In the case of the critical regime, *W*(*T*) is independent of temperature, and the slope of the *W*(*T*) plot is zero. The resistivity is not activated; however, the conductivity is given by a power law

*σ* (*T*) = *aT^β^* (7)

In the metallic regime, *W*(*T*) has a positive temperature coefficient and resistivity (ρ) is finite (*T*→0). Therefore, the electronic conductivity in the metallic regime is calculated by

(8) $$\sigma =\sigma_{0}+mT^{1/2}+BT^{p/2}\;\left(1.7\right)\;,\;m=\alpha \left[\frac{4}{3}-\gamma \left(\frac{3F_{\sigma }}{2}\right)\right]$$

where $\sigma_{0}$ is the zero-temperature conductivity, *B* is a constant depending on the localization effects, *α* is a parameter depending on the diffusion coefficient, *γF*_σ_ is the interaction parameter, and *p* is determined by the scattering rate (for electron-phonon scattering, *p* = 3; for inelastic electron-electron scattering, *p* = 2 in the clean (weakly disordered) limit or 3/2 in the dirty (strongly disordered) limit). The term “$mT^{1/2}+BT^{p/2}$” is mainly determined by the interaction and localization contributions to the conductivity [27,45,78]. In disordered materials, electron-electron interactions play an important role in transportation at low temperatures. The temperature dependence of conductivity in the three regimes of the metal-insulator transition for CPs has been reviewed in detail by Ahlskog et al. [77] (Table 4).

In general, the conductivity of doped CPs decreases with decreasing temperature, in contrast to the conductivity of conventional metals which increases with decreasing temperature. Precedent studies have found that the conductivity of CPs at higher doping levels shows weaker temperature dependence [79,80]. This characteristic suggests that the conductivity is still limited by phonon-assisted hopping between localized states resulting from material imperfections or tunneling between metallic regions [41]. The relationship between the DC conductivity and temperature for highly and moderately doped polyacetylene samples has been investigated by Roth et al. [81]. For a highly doped sample, the conductivity changes little with increasing temperature, while the change is dramatic for moderate doping levels. Aleshin et al. [82] reported the metallic behavior of PF_6_-doped PEDOT upon their studies of its temperature-dependent behavior. Its resistivity increased monotonically with falling temperature down to 10 K. When the temperature was lower than 10 K ($\rho_{r}=\left[\frac{\rho \left(1.4\;K\right)}{\rho \left(300\;K\right)}\right]<2.1$), the temperature coefficient of resistivity changed from negative to positive. This is a feature specific to conventional metals, and the transition can be explained by the contribution of electron-electron interactions at low temperatures. Recently, Lee et al. [83] discovered that camphor sulfonic acid (CSA)-doped PANI exhibits conductivities of more than 1000 S·cm^−1^ at room temperature, and that the resistivity exhibits the features of a conventional metal over a large temperature range below room temperature. The resistivity decreased as the temperature rose from 5 to 300 K with $\rho_{r}=\left[\frac{\rho \left(5\;K\right)}{\rho \left(300\;K\right)}\right]\approx 0.4$. This feature of CPs behaving like conventional metals over a large temperature range had never been reported in the literature before. These samples were on the metallic side of the insulator-metal transition. Thus, the use of these metallic polymers could be expanded to a range of important applications.

## 4. Electrochemical Properties

### 4.1. Reversible Oxidation/Reduction

As described in Section 2.2, doping typically leads to the formation of charge carriers, which is accompanied by changes (e.g., from benzenoid to quinoid) in the geometric structure of the CP. The original geometric structure can be recovered by reducing the polymer back to its pristine (undoped) state. The reversible doping/de-doping of the polymer corresponds to charge/discharge, which forms the basis of the principles behind polymer-based sensors and capacitors. The *p*-doping or electro-oxidation of CPs can be rationalized mechanistically as electrons in the π-bond being extracted and moving along the polymer skeleton while counter-anions from the electrolyte insert into the polymer chain to balance the electronic charge. The mechanism of *n*-doping or electro-reduction of CPs involves electrons being transported to the polymer backbone and counter-cations intercalating into the polymer backbone from the electrolyte solution in order to balance the overall charge.

**Figure 6:**
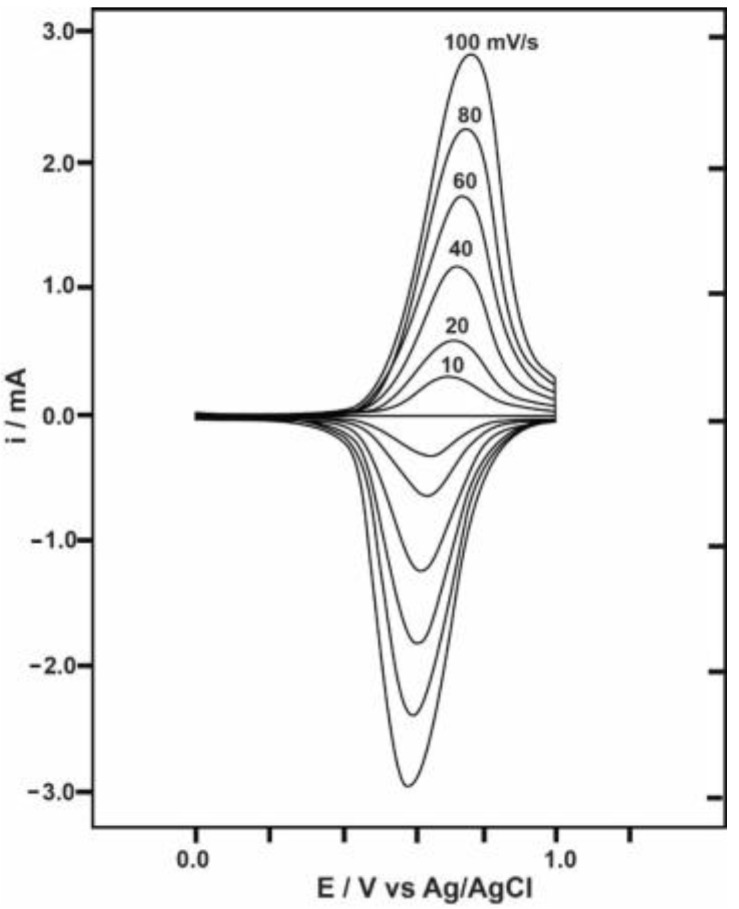
Cyclic voltammograms of a poly(*N*-phenyl-1-naphthylamine) film on Pt in 1 M LiClO_4_/CH_3_CN solution at different scanning rates. Reprinted with permission from [85]. Copyright 1990, American Chemical Society.

The most powerful electrochemical technique used to study redox processes in CPs is cyclic voltammetry (CV). CV measures the current resulting from an applied potential with a fixed scan rate (in mV·s^−1^). During redox reactions, reduction makes polymer chains negatively charged while oxidation produces positively charged polymer chains. When doping and de-doping are performed, ions move in and out of the polymer matrix. The current peak for a reversible system is then calculated by

(9) $$i=n^{2}F^{2}A\Gamma v\left[\frac{\exp\theta }{RT(1+\exp\theta )}\right]$$

where θ = (*nF*/*RT*)(*E* − *E*°), *n* is the number of electrons, and *A* is the electrode area (cm^2^). The current peak is directly proportional to the surface coverage (*Γ*) and potential scan rate (v), which is valid only for thin CP films with dopant ions that have small diffusion coefficients. This phenomenon has been discussed in studies on the electrochemical properties of BF_4_-doped PPy undertaken by Diaz et al. [84]. The cyclic voltammograms in such cases show completely symmetrical redox peaks (one-electron redox processes) in the range between the points on the curve at −0.4 and +0.3 V. The current increases proportionally with v and the voltammograms do not change when the solutions are stirred. The color of the film changes from yellow to black, corresponding to the neutral and oxidized states, respectively. With the use of very large or sluggish dopant ions for thick films, the electrochemical charging/discharging process becomes diffusion controlled. Current is then proportional to $v^{1/2}$ and the voltammogram changes from symmetrical to asymmetrical. It is clear that the reduction peaks shift negatively and the oxidation peaks shift positively with an increase in the scan rate (Figure 6) [85]. As the potential scan rate is increased, the voltage application becomes faster and the electrode material may not have enough time to undergo oxidation and reduction reactions completely. Hence, there may be severe kinetic limitations to charge transfer at high voltages.

**Figure 7:**
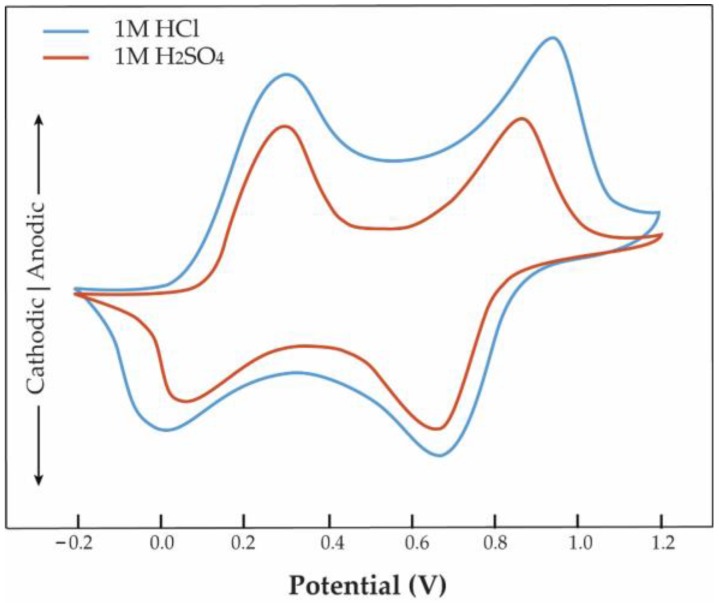
Cyclic voltammetry (CV) curves of a polyaniline (PANI) film doped with hydrochloric acid or sulfuric acid at the same potential scan rate 50 mV·s^−1^.

For multi-electron transfer processes, the cyclic voltammograms reveal several redox couple peaks. A typical voltammogram exhibiting reversible *p*-doping for PANI is shown in Figure 7. It shows that two pairs of peaks appear at a scan rate of 50 mV·s^−1^. Two oxidation peaks for PANI are observed at 0.72 and 0.31 V. The reverse scan features reduction peaks at 0.48 and 0.09 V corresponding to de-doping of the polymer. Interestingly, the cyclic voltammetric behavior of the PANI electrode is dependent of the kind of acid electrolyte used, which may be caused by the different sizes and charges of the dopants. Sulfuric and hydrochloric acid dissociate in water to produce protons and anions. Since protons are formed in both acids, the different cyclic voltammetric behaviors may be attributed to a difference between the ${\mathrm{SO}}_{4}^{2-}$ and ${\mathrm{Cl}}^{-}$ anions. The ionic radius increases with increasing number of electron shells. Therefore, the larger ion (${\mathrm{SO}}_{4}^{2-})$ is considered less diffused into the PANI electrode than the smaller ion (${\mathrm{Cl}}^{-}$). Similarly, the conductivity of the PANI can be also affected by the kind of dopant. PANI doped with hydrochloric acid exhibits higher electrical conductivity, indicating that the effective migration of anions (dopant ions) into the electrode material is a crucial factor affecting both the electrical and electrochemical characteristics of the CP electrodes.

**Figure 8:**
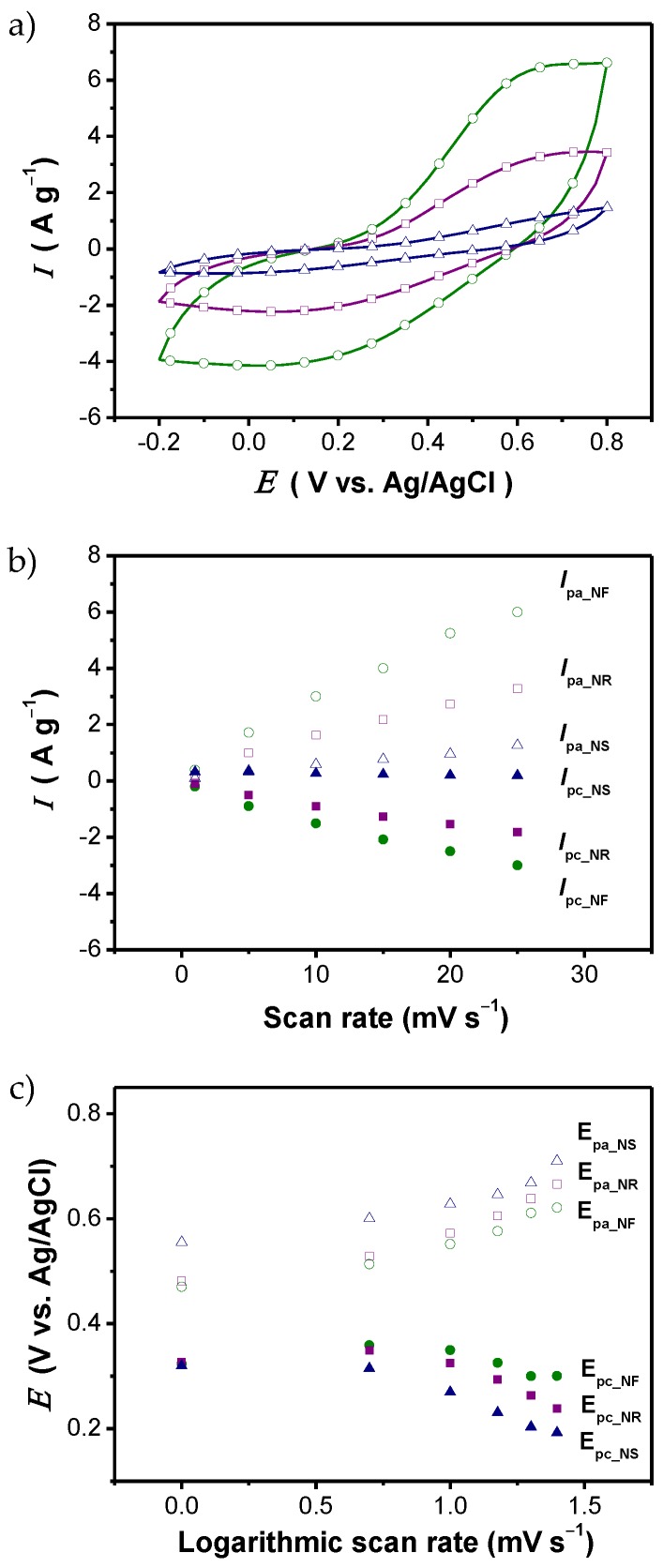
CV analysis of PANI nanostructures with three different shapes (nanosphere, NS; nanorods, NR; and nanofibers, NF) performed in a 1 M sulfuric acid solution. (**a**) Cyclic voltammograms of electrodes consisting of PANI nanostructures at the same scan rate (25 mV·s^−1^); (**b**) plots of the peak current (the anodic peak current, *I*_pa_; the cathodic peak current, *I*_pc_) vs. the scan rate; and (**c**) plots of the peak potential (the anodic peak potential, *E*_pa_; the cathodic peak current, *E*_pc_) vs. the log of the scan rate. With permission from [86]; Copyright 2012, American Chemical Society.

Doping processes clearly affect the electrochemical properties of CPs. It is also important to note that the morphology of a CP, particularly at the nanometer scale, can have a critical effect on its electrochemical properties. For example, Figure 8a shows the voltammograms of three different PANI nanostructures, i.e., nanospheres, nanorods, and nanofibers, recorded in sulfuric acid solutions (${\mathrm{SO}}_{4}^{2-}$ dopant anion) [86]. While the voltammograms all exhibit a similar shape, the integrated area of each voltammogram increases in the order nanospheres < nanorods < nanofibers. More specifically, the effect of the potential scan rate on the peak current was monitored, as seen in Figure 8b. Both anodic (*I*_pa_) and cathodic (*I*_pc_) peak currents are linearly proportional to the scan rate, indicating that electrode kinetics is subject to a surface-controlled redox process. Figure 8c shows the anodic (*E*_pa_) and cathodic (*E*_pc_) peak potentials as a function of the log of the scan rate. The electron transfer coefficient (α) and electron transfer rate constant (*k*_s_) can be calculated using the Laviron theory. The α values are found to be 3.6 × 10^−1^–3.7 × 10^−1^. The *k*_s_ value (4.3 × 10^−1^ s^−1^) of PANI nanofibers is higher than that of the other PANI nanostructures (nanorods: 3.1 × 10^−1^ s^−1^; nanospheres: 2.6 × 10^−1^ s^−1^), indicating that the electron transfer capability of PANI is highly dependent on the structural characteristics.

The redox processes of CPs are stable and reversible in an established potential range. However, when a CP electrode is scanned at higher potentials, the CP undergoes rapid structural degradation and loss of electroactivity (irreversible oxidation). These phenomena have been reported to be because of so-called overoxidation [87]. The mechanism of overoxidation is quite complex and the irreversible structural change upon overoxidation is not fully understood. Beck et al. [88,89] found that overoxidation is strongly influenced by the presence of nucleophiles such as OH^−^ and Br^−^. In other studies, overoxidation also showed a dependence on the pH of the electrolyte. In some cases, no detectable overoxidation occurred at pH < 0.1 [90,91]. Therefore, the choice of solvent and electrolyte as well as the electrochemical potential play important roles in avoiding irreversible structural change during the application of CPs.

### 4.2. Pseudocapacitance

Recently, significant attention has been dedicated to the application of CPs in electrochemical capacitors. Electrochemical capacitors can be divided into two device types according to the mechanism of charge storage: electrochemical double layer capacitors (EDLCs) and pseudocapacitors. EDLCs are based on non-Faradaic phenomena. They store charge using reversible adsorption of electrolyte ions onto large-surface-area electrodes. Various materials have been extensively examined and utilized for EDLCs. Representatively, porous carbon materials with high surface-to-volume ratios have been widely used as electrode materials for EDLCs [92]. In contrast, charge storage in pseudocapacitors is based on a Faradic mechanism, which takes advantage of redox reactions to store charge in the electrode. This feature makes pseudocapacitors exhibit battery-like behavior in charge/discharge processes. In general, pseudocapacitors provide higher specific capacitance and energy density than EDLCs. The reversible redox capability of CPs allows pseudocapacitive charge storage. During charging, anions from the electrolyte are inserted into the polymeric chain and electrons are released from the CP. In other words, the charging is accompanied by an oxidation process. Conversely, discharging proceeds through a reduction process. For instance, the charge/discharge process for PANI in sulfuric acid solution (*p*-doping) can be expressed as follows:

$$\text{Charging}\;\text{process}\;(\text{oxidation}):\;\mathrm{PANI}+n{\mathrm{SO}}_{4}^{2-}\to {\mathrm{PANI}}^{2n+}:n{\mathrm{SO}}_{4}^{2-}+2ne$$

$$\text{Discharging}\;\text{process}\;(\text{reduction}):\;{\mathrm{PANI}}^{2n+}:n{\mathrm{SO}}_{4}^{2-}+2ne\to \mathrm{PANI}+n{\mathrm{SO}}_{4}^{2-}$$

To study the electrochemical performance of pseudocapacitors, CV may be performed in a three-electrode cell to determine the capacitance. Under redox cycles, the specific capacitance is obtained from CV curves according to the following equation [93]:

(10) $$C=\frac{1}{mv\left(V_{b}-V_{a}\right)}\int_{b}^{a}idV$$

where *i* is the discharge current corresponding to the reduction peak in the case of *p*-doping (and the oxidation peak in the case of *n*-doping), v is the potential sweep rate, *m* is the mass of deposited material, and *V*_a_ and *V*_b_ are the high and low potential limits of the CV tests, respectively.

The mechanical stress in CP films has already been proven to be associated with the cycle life of pseudocapacitors. Hu et al. descried the cycle stability of HCl-doped PANI film in 1 M H_2_SO_4_ at a rate of 20 mV·s^−1^ [94]. The reduction peak had nearly disappeared by the 500th cycle, which indirectly implies instability of the capacitor electrode. Upon redox switching, volume change occurs because of insertion/extraction of dopants into and from the polymer chain. The large volumetric swelling, shrinkage, and cracking of CPs during charge/discharge (doping/de-doping) processes often lead to mechanical deterioration of the polymer structure, degradation of electrochemical performance, and rapid decay of capacitance [95]. Thus, achieving long-term cycle stability is a significant challenge for high-performance pseudocapacitors based on CPs. Conversely, the performance of energy storage devices including pseudocapacitors is evaluated mostly by measuring energy density and power density. The energy density is the maximum energy that a device of a given size or mass can store. The power density measures how quickly the battery can deliver energy for a given size. The power density (*P*) and energy density (*E*) of a CP electrode material are normally derived from galvanostatic charge/discharge cycles and can be calculated using the following equations:

(11) $$E=\frac{1}{2}C\Delta V^{2}$$

(12) $$P=\frac{E}{\Delta t}$$

where C=iΔt/mΔV is the specific capacitance derived from galvanostatic tests, ∆*t* is the discharge time, and ∆*V* is the potential window (or the voltage range for two electrode cells). To enhance the power density and energy density of pseudocapacitors, it is very important to maximize the specific capacitance and potential window. Currently, most studies are focused on developing advanced materials to improve the conductivity, charge/discharge rate, effective surface area, and redox capability of pseudocapacitors. For example, CPs have been combined with other functional materials, which has provided opportunities for ameliorating conductivity (particularly at the more negative/reducing potentials), cycling stability, mechanical stability, specific capacitance, and processibility [96,97].

### 4.3. Swelling and De-Swelling

**Figure 9:**
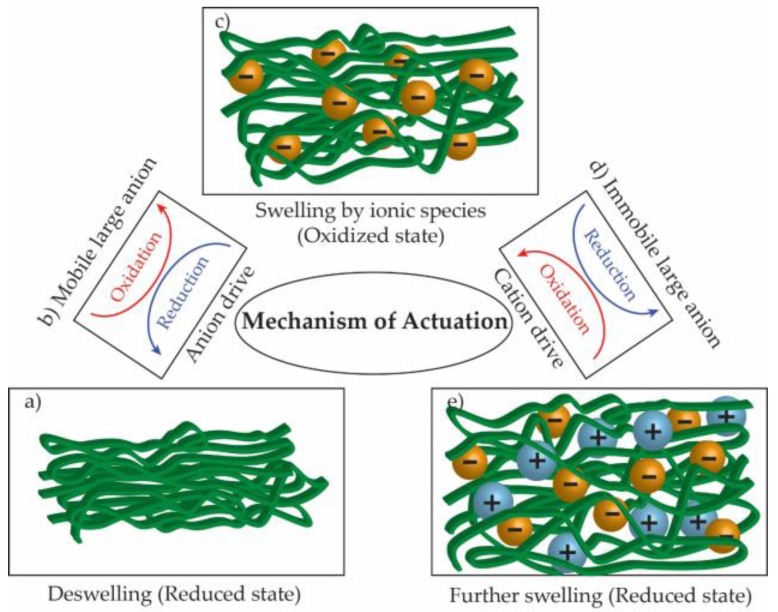
Mechanism of electrochemo-mechanical actuation in CPs. (**a, c, e**) Volume changes in CP via (**b**, **d**) two different redox pathways.

The insertion/de-insertion of ions/solvent to CP matrix during redox processes can lead to extensive changes in the dimensions of the polymer [98,99,100]. CPs undergo swelling and de-swelling (volumetric change) as their redox state is changed. This swelling/de-swelling can be separated into two components: an intrinsic part due to changes in bond lengths and conformation of the polymer backbone, and osmotic expansion of the polymer phase [101]. This phenomenon has been proposed to be exploitable for a new generation of actuators. CP actuation can be chemically and electrochemically controlled [102]. Representatively, the mechanism of the electrochemical actuation in CPs is illustrated in Figure 9. Polymer chains become positively charged when electrons are extracted by oxidation. Small anions are inserted into the polymer matrix in order to maintain overall charge neutrality. Ionic crosslinks are formed between polymer chains and anions, resulting in an increase in the total volume. The dopant anions can be expelled during electrochemical reduction by the application of negative voltages (Figure 9a–c). Essentially, this anion-driven actuation causes swelling upon oxidation and de-swelling upon reduction [103].

In other cases, when the anions that are incorporated during polymerization are large enough, they become immobilized and are thus trapped inside the polymer structure. The CPs then show further swelling on addition of cations from the solution for charge compensation during reduction (Figure 9c–e). This type of actuation can be defined simply as cation-driven actuation. Electrochemical quartz crystal microbalance studies have shown that the expansion decreases as the electrolyte concentration increases. The total volume change caused by solvent molecules and ions can be explained by the osmotic effect [101]. Free-standing films of PPy doped with dodecylbenzene sulfonate were investigated to examine the effects of the solvent molecules and electrolyte ions. As the electrolyte concentration increased from 0.1 to 1.0 M, the expansion decreased by around 30%. This result agrees with the observations made by Maw et al. [104] and Aydemir et al. [105], which indicated that solvent molecules move in and out of the film associated with the electrolyte [99]. There are many significant parameters affecting this actuation, such as the chemical/physical properties of the CP and the size/type of dopant and electrolyte ions. In addition to tailoring the properties of the main components, such the CP and electrolytes, it is very important to design efficient actuating systems for significant improvement of the performance of CP-based soft actuators. Several remarkable actuating systems have been designed, such as bilayer-trilayer actuators, out-of-plane actuators, and linear actuators [102].

### 4.4. Electrochromism

It is known that reversible doping/de-doping of CPs is mediated by oxidation/reduction processes. Visible color change of CPs can be effected by reversible redox processes, a phenomenon known as electrochromism. This interesting property makes CPs attractive candidates for electrochromic applications such as electrochromic displays [106], smart windows [107], and rearview mirrors. It is believed that this color change depends on both the energy gap of the CPs and the dopants. The mechanism of electrochromism is due to insertion/de-insertion of dopant ions through doping/de-doping processes. The doping induces reorganization of the electronic structure of the polymer, resulting in reduced energy gap for the π–π* transition. The formation of sub-bands by charge carriers such as polarons and bipolarons modulates the absorbance of the CPs, which causes changes in their color. Normally, the energy gap for pristine CP thin films is higher than 3.0 eV, and the films are colorless and transparent in the undoped state. Thin films can also show the strongest absorption spectra in the doped state in the visible region. If pristine CPs have an energy gap around 1.5 eV, they strongly absorb visible light and produce high-contrast colors in the undoped state. However, after doping, the absorption wavelength moves to the near-infrared region [107].

**Figure 10:**
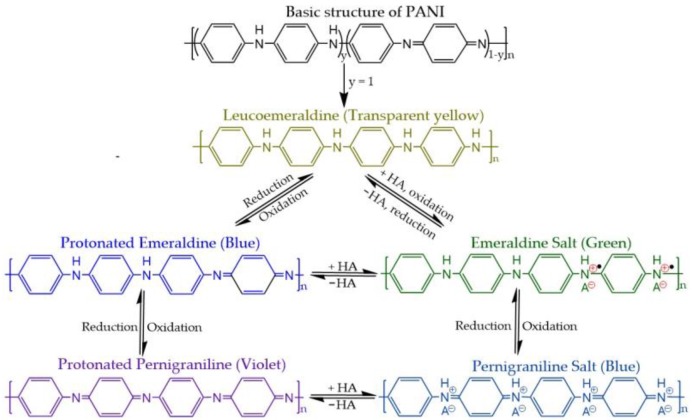
The different redox/protonation states and colors of PANI.

The colors of CPs may be different in different redox states. For instance, electrochromic properties of PANI (*p*-doping) have been widely studied in acid, salt, and organic media by CV. Various forms of PANI related to pronation/deprotonation and insertion/de-insertion of anions have been reported. Nitrogen atoms in PANI chains are responsible for the injection of protons or anions to form radical cations [108,109,110]. The different redox/protonation states of PANI are presented in Figure 10. PANI exhibits color changes from transparent yellow to green, blue, and violet. The absorption spectra can be extended from the visible to the infrared region using the complexation of CPs [111]. The electrochromic properties of CPs depend on chemical structures, redox capability, temperature, and the pH of the electrolyte solution. In particular, the switching time of the color changes strongly depend on the speed of migration with which protons/dopant ions move in and out of the polymer matrix. Therefore, the morphology and microstructural properties of CPs, such as their dimensions and porosity, should be controlled to enhance the rate of color change. For extended application of CPs to electrochromic devices, it is necessary to further enhance their properties to ensure long life cycle time, rapid color change switching, and high color contrast.

## 5. Applications

### 5.1. Electrochemical Capacitors

Pseudocapacitive CPs exhibit high conductivity, structural flexibility, and chemical stability in corrosive electrolytes. In this section, the development of CP-based pseudocapacitive materials is reviewed in reference to the remarkable studies undertaken recently [112,113].

**Figure 11:**
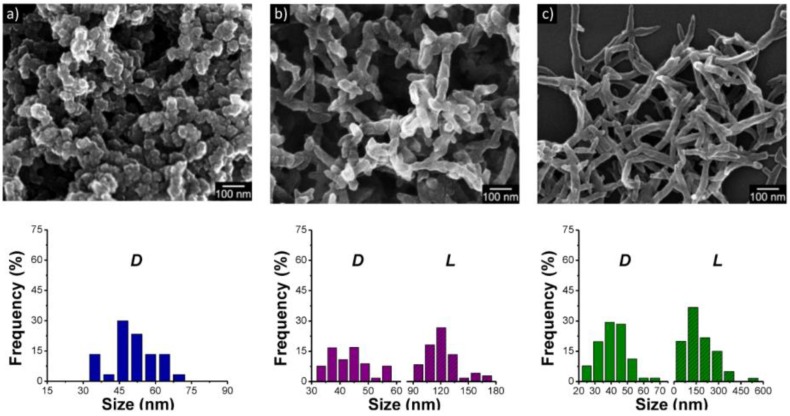
Field-emission scanning electron microscope images of PANI nanostructures with different aspect ratios synthesized under the same stirring conditions (200 rpm) and histograms showing their size distribution (*D*, diameter; *L*, length): (**a**) nanospheres; (**b**) nanorods; and (**c**) nanofibers. With permission from [86]; Copyright 2012, American Chemical Society.

The electrode materials most widely used for pseudocapacitors are CPs and metal oxides such as MnO_2_ [114] and NiO [115]; metal chalcogenides such as TiS_2_ [116], MoS_2_ [117], and Co_0.85_Se [110]; and metal hydroxides such as CoSn(OH)_6_ [118]. In particular, metal oxides have been intensively studied for pseudocapacitors because they exhibit high theoretical capacitances. However, their poor conductivity, inflexibility, and high cost prevent them from being widely applied. In contrast, CPs such as PANI and PPy not only show high energy storage capacity but also present the possibility of fabricating flexible electrodes. However, a serious drawback of CPs in practical application lies in their poor cycling stability. To resolve this issue, many researchers have developed new methods for preparing electrode materials with improved microstructures and morphologies based on hybridization and the fabrication of well-designed nanostructures. These materials show enhanced cycling stability, power density, and energy density. As an interesting example, the relationship between the morphology of PANI nanostructures and their capacitance has been investigated by Park et al. [86]. PANI nanospheres were synthesized in an acidic aqueous solution. They were then evolved into nanorods and nanofibers by varying the concentration of the steric stabilizer. The morphologies of the PANI nanostructures are exhibited in Figure 11. The discharging capacitance and cycling stability of the three different nanostructures were examined under the same conditions. The specific capacitances were measured to be 71, 133 and 192 F·g^−1^ for nanospheres, nanorods, and nanofibers, respectively. In other words, the higher the aspect ratio of the nanostructure, the higher the specific capacitance. This is because the oxidation/protonation level of PANI increased with increasing aspect ratio. Better structural ordering in the PANI chains is believed to contribute to the outstanding oxidation level of the nanofibers. Furthermore, the nanofiber electrode had faster electrode kinetics than the nanorod and nanosphere electrodes. Thus, the morphology or aspect ratio of the nanostructure is a significant factor affecting the performance of the related pseudocapacitor. A year later, Chen et al. [119] continued to investigate PANI nanostructures of different shapes (i.e., nanofibers, nanospheres, and nanotubes) prepared using MnO_2_ reactive templates for pseudocapacitor applications. The nanotubes showed the best capacitance owing to its higher surface area. In a redox active electrolyte, the nanotubes showed an improved specific capacitance of 896 F·g^−1^@10 A·g^−1^, which is the maximum value achieved for a PANI capacitor cell reported so far. Finally, the cycling stability of the PANI nanotubes over 5,000 cycles was much better than those of the other two nanostructures [120,121,122].

**Figure 12:**
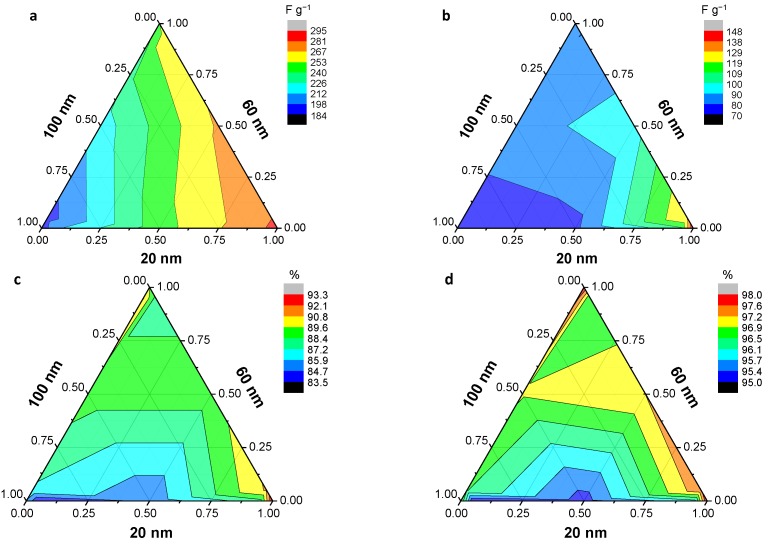
Effect of binary nanoparticle packing on electrode performance. Ternary diagrams for nanospheres of three different diameters showing the distribution of (**a**,**b**) specific capacitance and (**c**,**d**) coulombic efficiency as a function of the mixed weight fraction (*f*_m_) measured at (**a**,**c**) 0.1 A·g^−1^ and (**b**,**d**) 1.0 A·g^−1^. A three-electrode system was used with 1 M sulfuric acid solution. The *E*_c_ values were calculated from the charge/discharge curves. Reprinted with permission from [123]. Copyright 2016, Royal Society of Chemistry.

The dependence of pseudocapacitance on the size of CP nanoparticles has also been investigated. It was expected that the size of the CP nanoparticles would proportional to the pseudocapacitance because the redox process is mostly confined to the surface of the polymeric nanoparticles. However, nanoparticles must pack together to form bulky electrodes for applications, and the packing of the nanoparticles significantly affects the performance of these pseudocapacitive electrodes. Lee et al. [123] examined the random packing effect for binary mixtures comprising two of three different diameter PPy nanospheres (20, 60, and 100 nm) on the pseudocapacitance of macro-scale electrodes. The packing of the binary nanosphere mixtures showed diverse and complicated behavior in terms of effective surface area and porosity. Importantly, they found that the use of nanosphere mixtures with lower diameter ratios (20 nm/100 nm) at an optimized composition led to more dense and efficient packing, which in turn contributed to better device performance (enhanced specific capacitance and coulombic efficiency) (Figure 12). Although PPy is a soft material, as mentioned earlier, most of the faradaic redox reaction for pseudocapacitance is confined to the PPy surface. Therefore, geometrical factors like the surface area and porosity depending on the packing of nanospheres could determine the performance of the nanosphere-assembled electrodes.

**Figure 13:**
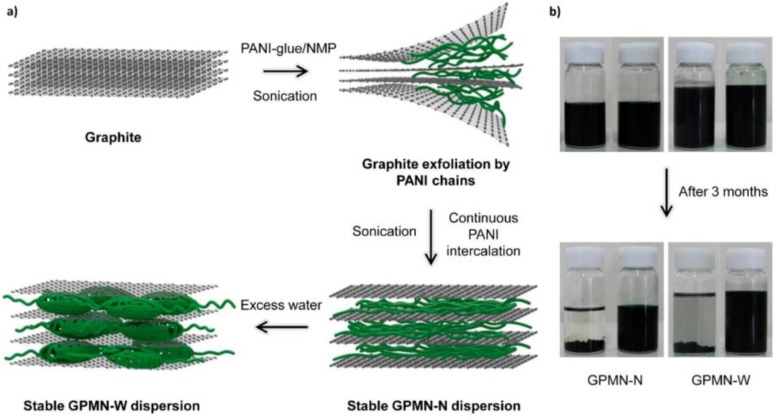
(**a**) Schematic illustration of the formation of graphene/PANI multilayered nanostructures (GPMNs) by direct physical exfoliation of graphite with PANI glue; (**b**) photographs showing the long-term colloidal stability of a GPMN dispersion solution in the absence (**left**) and presence (**right**) of PANI glue. GPMNs showed outstanding colloidal stability in both NMP and water. Reprinted with permission from [126]. Copyright 2015, John Wiley & Sons.

However, it has been noted that control of the size and morphology of a CP material does not improve its performance beyond an intrinsic limit. A number of heterogeneous components have been introduced into CPs to overcome this intrinsic limit. For example, a combination of three organic additives (d-glucitol, dodecylbenzenesulfonic acid, and CSA) has been used as a complex dopant for improving the adhesive properties and conductivity of PANI [124]. Hybridizing CPs with carbon nanomaterials is also believed to improve the electrical, electrochemical, and mechanical properties of the corresponding electrodes [125]. Hybrid nanomaterials can be obtained by physical and chemical methods. For instance, Choi et al. [126] have described a simple physical method to prepare graphene/PANI multilayered nanostructures (GPMNs) for flexible electrochemical capacitor application. PANI was spontaneously intercalated between the graphene sheet layers without deterioration of the sp^2^-hybridized bonding structure under sonication (Figure 13). Compared with PANI alone, the GPMNs exhibited greatly improved charge/discharge cycling stability, retaining 82% of their initial specific capacitance after 5000 cycles. Graphene imbued the material with prodigious mechanical properties and aided formation of the charge-transfer complex, which mitigated the rapid capacitance degradation of PANI caused by volume changes or mechanical issues. Interestingly, the micromorphology of the PANI between the graphene layers was highly dependent on the dispersion medium. The GPMNs were produced from *N*-methyl-2-pyrrolidone (NMP) (GPMN-N). When the NMP was replaced with water (GPMN-W), the PANI chain agglomerated to form larger granular clusters. GPMN-Ws had a more porous structure, leading to different electrochemical properties. Yu et al. [127] showed that reduced graphene oxide (RGO)-sheet-wrapped PANI nanowire arrays grown on a nitrogen-doped carbon fiber cloth (NCFC) exhibited excellent mechanical, physical, and chemical properties. The RGO layer accommodated volume changes and mechanical deformation of the coated PANI nanowire arrays during long-term charge/discharge processes. The RGO/PANI/NCFC composite electrode had a large surface area and superior conductivity, providing a high specific capacitance of 1145 F·g^−1^. Furthermore, this material retained over 94% of its initial capacitance after 5,000 cycles. The use of carbon nanomaterials is extremely interesting. Consequently, many hybrids such as PANI/carbon nanotubes [128], PPy nanowires/carbon cloth [129], carbon nanofiber/graphene oxide/PANI [130], PPy/rGO and PANI/rGO bilayers [131], PANI/graphene nanosheets [132], and PANI or PPy/carbonaceous films [133] have been reported.

**Figure 14:**
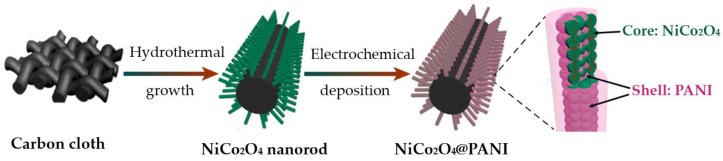
Schematic of the synthetic procedure for NiCo_2_O_4_@PANI nanorod arrays. Reprinted with permission from [134]. Copyright 2016, American Chemical Society.

To obtain improved properties, a large number of recent works regarding CP nanocomposites have focused not only on using carbon nanomaterials but also on using metal compounds. For instance, CPs may be coated onto metal nanostructures to improve their electrochemical performances. A heterojunction structure composed of a conductive metal core and a nanoscale CP sheath presented enhanced specific capacitance and maintained good cycling stability [134]. Jabeen et al. [134] combined PANI with mesoporous NiCo_2_O_4_ (NiCo_2_O_4_@PANI) into a hybrid electrode material. Figure 14 shows that NiCo_2_O_4_ nanorod arrays were first grown on the carbon cloth by a hydrothermal process, followed by coating PANI on the NiCo_2_O_4_ by electrodeposition at an applied potential of 0.8 V (vs. Ag/AgCl). The products possessed a unique core–shell heterostructure that was favorable for the full exertion and utilization of the pseudocapacitive PANI layer. In addition, the porosity of the NiCo_2_O_4_ facilitated ion transport and accommodated structural deformation of the hybrid electrode. Consequently, the electrode material exhibited a high specific capacitance of 901 F g^−1^ and an outstanding cycling stability of approximately 91% after 3,000 charge/discharge cycles. As another recent example, PANI-coated hexagonal molybdenum trioxide (h-MoO_3_) hollow nanorods were obtained through a bottom-up approach [135]. Briefly, h-MoO_3_ hollow nanorods were formed using the cation-exchange-assisted Kirkendall effect. Chemical oxidation of aniline monomers on the active h-MoO_3_ surface resulted in the formation of a PANI sheath. Compared with PANI or h-MoO_3_ hollow nanorods alone, the combination of the heterogeneous redox-active components (PANI@h-MoO_3_) reinforced the pseudocapacitive performance of the nanocomposites over a wide range of current densities. The hollow structures can accommodate higher strain produced by a large volume change during the charge–discharge process. Moreover, the presence of a PANI shell around the h-MoO_3_ core could significantly improve the cycling stability of the nanocomposite electrodes. Therefore, the nanocomposite electrode showed an improved specific capacitance maximum of 270 F·g^−1^ when compared to that of the pristine h-MoO_3_ hollow nanorods (126 F·g^−1^) and PANI only (180 F·g^−1^), and enhanced cycling stability.

Furthermore, the performance of CP-based electrochemical capacitors also depends on the parameters of the capacitor cells, such as the symmetric/asymmetric electrode configuration, membrane, electrolyte, and electrode mass ratio. Therefore, it is highly important to optimize the cell system for practical applications.

### 5.2. CP Sensors

A sensor is a device that converts an input parameter (e.g., temperature, humidity, or chemical and biological species) into a signal that can be measured. The output signal of the sensor is normally based on changes in electrical or optical properties. CPs show reversible changes in conductivity, color, and volume, which makes them suitable materials for sensing. Since CPs can interact with organic compounds and moisture present in the surrounding environment, they are sometimes unstable and consequently may exhibit low sensitivity and selectivity to a target analyte, which needs to be improved. There have been many efforts to improve the sensitivity and selectivity of CP sensors by tailoring their properties [22,136,137]. A variety of functional heterogeneous materials have been introduced to CPs for modifying their electronic, chemical, and microstructural properties, resulting in interesting findings [125,137]. Regarding heterogeneous functional components, current interests focus on use of chalcogenides, carbon nanotubes (CNTs), graphene, metal oxide particles, metal particles, and other polymers. CP sensors have been realized by exploiting conductometric, potentiometric, amperometric, voltammetric, gravimetric, pH-based, and incorporated-receptor-based sensing modes [22,138,139].

#### 5.2.1. Chemical Sensors

First-generation pristine CP film sensors (un-doped) were used to detect gas/vapor species (including NO_2_, SO_2_ and I_2_) by directly exposing the sensors to the target environment and monitoring changes in conductivity. However, this method faced the limitation of poor selectivity because the surrounding environment normally contains many interferences including humidity. Forzani et al. [140] developed a chemical sensor based on both amperometric and conductometric modes. The dual-mode sensor used PANI to detect an analyte through both individual and simultaneous changes in the electrochemical current and conductance of the polymer. This sensor showed improved selectivity for the detection of target analytes in complex samples and even in the presence of high concentrations of unexpected elements by control of the applied potential. Many researchers have developed metal/CP nanocomposites for rapid, selective CP chemical sensors. Athawale et al. [141] used a Pd/PANI nanocomposite as a component for selective methanol sensors. The resulting nanocomposite was highly sensitive and selective to methanol vapors in the air. Fourier transform infrared spectroscopy analysis demonstrated that the Pd nanoparticles catalyzed the reduction of the imine nitrogen of PANI to an amine by methanol, contributing to the enhanced selectivity of the nanocomposite. Various metal or metal compounds/CP nanocomposites have been employed to detect different chemical targets, as listed in Table 5.

To enhance the performance of CPs in chemical sensors, Kwon et al. [148] investigated the effect of PPy nanoparticle size on chemical sensing behaviors in the detection of volatile organic compounds and toxic gases. PPy nanoparticles with diameters of 20, 60 and 100 nm were chemically fabricated using a soft-template approach in aqueous solution. It was found that the conductivity and surface-to-volume ratio increased with decreasing nanoparticle sizes. The chemical sensor based on the smallest PPy nanoparticles (20 nm) showed the best sensing performance, i.e., excellent sensitivity, rapid response time, reversibility, and reproducible responses. A large surface area promotes effective contact with analytes, which leads to high sensing performance. Furthermore, multidimensional PPy nanotubes with surface substructures have been fabricated and used to develop highly sensitive and selective chemiresistive sensors for monitoring volatile organic compounds and toxic gases in human breath. The lowest detection limit was 10 ppm for gaseous ammonia, which is the most sensitive recognition of ammonia by such sensors so far. The important point in this discovery is that the unique morphology of the multidimensional nanotubes was advantageous for sensor application by virtue of their improved charge transport behavior and enlarged effective surface area [149,150]. Furthermore, PPy nanotubes functionalized with carboxylic acid were used to fabricate a chemiresistive gas sensor for detecting dimethyl methylphosphonate, a nerve agent [151]. The presence of carboxylic groups on the surface of PPy nanotubes through intermolecular hydrogen bonding enhanced their sensitivity to dimethyl methylphosphonate. The result showed that the sensitivity of the chemiresistors was strongly dependent on the degree of functionalization with the carboxylic group.

**Figure 15:**
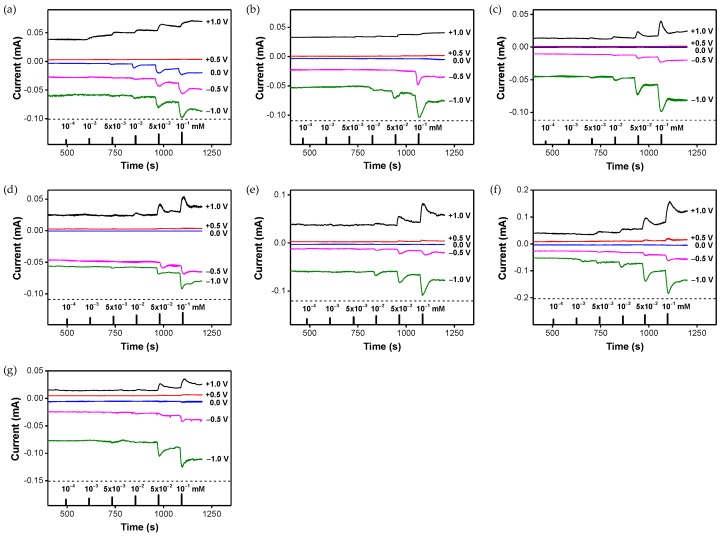
Real-time response of PPy/cellulose (PPCL) composite membranes in a flow cell measured at different applied potentials: (**a**) Hg(II); (**b**) Ag(I); (**c**) Pb(II); (**d**) Ni(II); (**e**) Cd(II); (**f**) Cr(III); and (**g**) Zn(II). Reprinted with permission from [152]. Copyright 2014, Royal Society of Chemistry.

**Figure 16:**
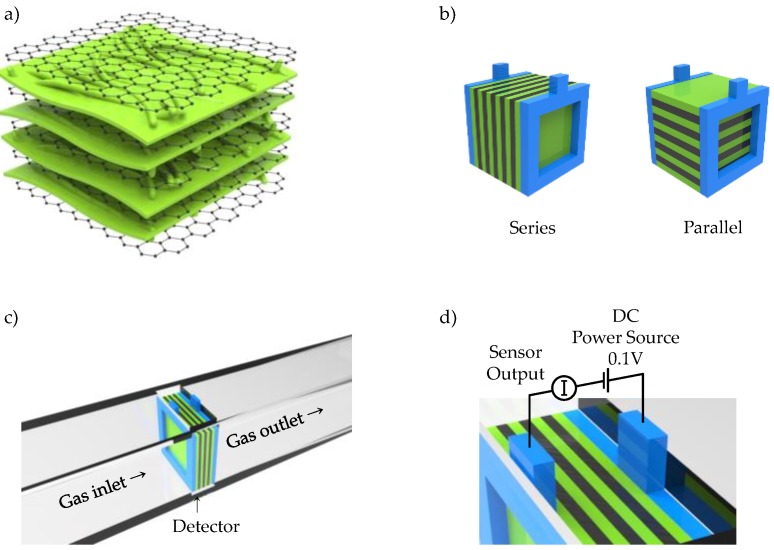
Schematic illustrating (**a**) the alternating layered structure of graphene/PANI; (**b**) the series connection and parallel connection-like structures formed by different orientations of the graphene/PANI film between the electrodes; and (**c**,**d**) the resistometric sensor setup for measuring the electrical response of the graphene/PANI films (electrode, blue; PANI, green; graphene, black). Reprinted with permission from [153]. Copyright 2016, American Chemical Society.

Hybridizing carbon nanomaterials with CPs is not only popular in electrochemical capacitors but also has applications in sensing applications. PPy/cellulose (PPCL) composite membranes have been demonstrated to be a potential material for detection and removal of metal ions in flow systems [152]. PPy was coated onto a cellulose membrane without any significant loss of microstructural integrity via vapor deposition polymerization. In the resulting PPCL composite membranes, PPy gave excellent electrochemical properties while cellulose provided high mechanical strength for enduring solution flow. Remarkably, the PPCL membranes revealed different electrochemical properties under different applied potentials, which led to differentiable signal responses and adsorption efficiencies, even in the flow system (Figure 15). Principal component analysis, a statistical technique for finding covariances among multivariate data, was used to study the ability of the PPCL membrane to discriminate between seven metal ions. In particular, the PPCL composite membranes exhibited high recognition and enormous adsorption efficiency for Hg(II), Ag(I) and Cr(III). This result implies that the sorption of metal ions on the PPCL membrane influences the electrochemical properties of the membrane, leading to the change in current. The composite membranes showed unique signatures for the three metal ions even in a real sample (groundwater). Recently, random layer-by-layer graphene/PANI films were exploited as transducer electrodes to detect NH_3_ gas based on resistometric action (Figure 16) [153]. As illustrated in Figure 16b, a series or parallel connection-like configuration of intercalated PANI layers was made when a voltage was applied perpendicularly or parallel to the stacked graphene plane. The graphene/PANI film contained numerous interfacial contacts between the highly conductive graphene and the semiconductive graphene, which provided unique anisotropic properties. Interestingly, the series connection-like configuration exhibited better sensing performance, particularly in terms of sensitivity. With an increase in demand for flexible, low-cost, and environmentally friendly devices, these fascinating studies on hybridizing carbon nanomaterials and CPs provide excellent opportunities for the next generation of sensors.

#### 5.2.2. Biosensors

**Figure 17:**
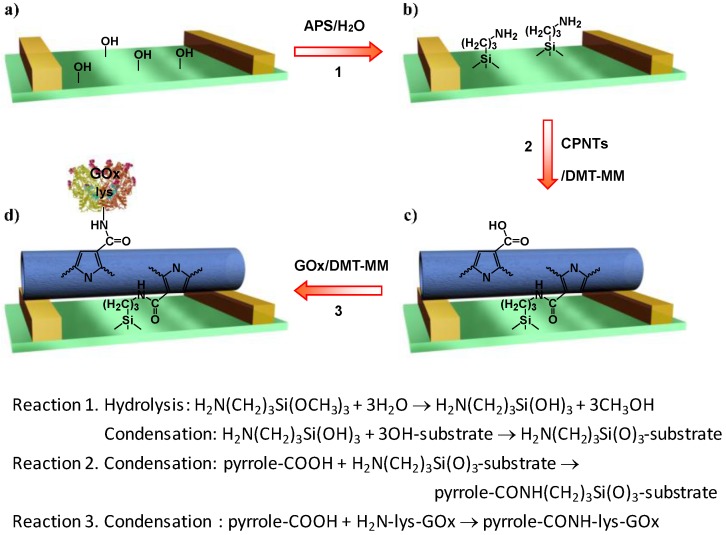
Schematic illustration of the reaction steps for the fabrication of a field-effect transistor (FET) sensor platform based on carboxylated PPy nanotubes (CPNTs): (**a**) microelectrode substrate; (**b**) aminosilane-treated substrate; (**c**) immobilization of the nanotubes onto a substrate; and (**d**) binding of GO_x_ to the nanotubes. Reprinted with permission from [154]. Copyright 2008, American Chemical Society.

A rise in the number of people affected by disease has prompted researchers to develop suitable devices for the fast and accurate diagnosis of biological target species. A number of researchers have used CPs and CP composites for biosensor applications because of their high compatibility with biological systems. Yoon et al. [154] developed a glucose biosensor based on enzyme-functionalized PPy nanotubes using a liquid-ion gated field-effect transistor (FET) configuration. Compared with 2D materials, 1D CP nanomaterials as the conductive channel of FETs can provide more sensitive responses through depletion or accumulation of charge carriers in the nanomaterial bulk. The CP nanomaterials should be immobilized on electrode substrates for sensing applications in the liquid phase. However, owing to possible chemical, thermal, and kinetic damages, CPs are unsuitable for conventional lithographic process. Fortunately, the CP nanomaterials could be chemically immobilized onto a surface-modified microelectrode substrate. Figure 17 depicts the detailed surface modification steps for fabricating the FET sensor substrate. In the first step, carboxylated PPy nanotubes (CPNTs) were anchored onto a microelectrode substrate through covalent linkages and contacted to two metal electrodes (called the source and the drain). Subsequently, the surface of the CPNTs could be functionalized with the enzyme glucose oxidase (GO_x_) through covalent binding. When the enzyme recognizes glucose, it induces a change in the source-drain current. This FET sensor showed high sensitivity in detecting glucose at the concentration range 0.5–20 mM. A similar concept has also been applied to detecting different kinds of biomolecules such as proteins [155,156], hormones [157], tastants [158] and odorants [159].

Three-electrode electrochemical configuration is also a common strategy for the fabrication of CP-based biosensors. A gold/PANI nanocomposite was reported for the electrochemical detection of prostate-specific antigen (PSA) [160]. The hybrid nanocomposite was obtained via electrostatic self-assembly of gold nanoparticles on PANI nanowire-deposited electrodes. The nanostructured gold/PANI composite provided effective surface sites for the immobilization of anti-PSA that facilitated charge transport and thereby enhanced sensing properties. The immunochemical sensor measured the concentration of PSA by differential pulse voltammetry. This sensor not only exhibited a long linear range in a calibration curve with a detection limit of 0.6 pg·mL^−1^, but also showed high sensitivity, selectivity, and reproducibility in response. Recently, Hui et al. [161] developed an electrochemical DNA sensor based on polyethylene glycol (PEG)ylated PANI nanofibers for breast cancer susceptibility gene (BRCA1) detection. PEG is an inexpensive, nontoxic, and highly hydrophilic polymer; therefore, it has been widely used in versatile applications from industrial manufacturing to medicine. PEGylation involves covalently coupling a PEG structure to another larger molecule. Remarkably, the PEGylated PANI nanofibers had large surface area and remained conductive. Additionally, they showed excellent antifouling performances in single protein solutions as well as in complex human serum samples. Consequently, the DNA sensor showed a very high sensitivity to BRCA1 with a linear range of 0.01 pM to 1 nM, indicating the excellent potential of this novel biomaterial for application in biosensors.

## 6. Conclusions and Outlook

We have described the electrical and electrochemical studies of CPs that have become important over the last few decades. Understanding these properties is of key importance in the development of CPs for application in various fields. The theoretical modeling of the transport properties of CPs is still challenging due to the extreme complexity of the polymeric conjugated systems. However, continuous study of the origins of conductivity and knowledge of doping reactions have made it possible to control the kinetics of the charge-transfer reactions. Electrons are either added to or extracted from the delocalized π-bonded polymer backbone, leading to the formation of charge carriers such as polarons, bipolarons, and solitons. The mobility of these carriers is affected by a variety of factors, such as the dopant, temperature, and inherent structure. The conductivity can be improved successfully by electrochemical doping, both *n*-type and *p*-type, to induce an insulator-to-metal transition in the CPs. CPs offer many advantages in applications as in electrochemical capacitors, actuators, and sensors. However, the major disadvantages of CPs when used in these applications include their inferior long-term stability. Furthermore, the conductivity of CPs still has much room for improvement. The conductivity of CPs is lower than that of metals, and this has limited their use in specific applications such as transistors and memory devices. It is believed that overcoming these existing problems will make CPs strong candidates for a diverse range of future applications.

## Figures and Tables

**Table 1:** CPs in which a maximum metallic state has been observed in the doped state [45].

CP	Repeat Unit	Chain Orientation	Conductivity (S·cm^−1^)
Polyacetylene	C_2_H_2_	High	10^4^–10^5^
PPV	C_6_H_4_–C_2_H_2_	High	10^4^
PPy	C_5_H_2_N	Low	400
PANI	C_6_H_4_–NH	Low	400
Poly(3-methylthiophene)	C_5_H_2_S–CH_3_	Low	400
PEDOT	C_7_H_4_O_2_S	Low	300

**Table 2:** Typical examples of dopants for CPs and the conductivities obtained therefrom.

CP Type	Dopant	Chemical Source	Doping Method	Conductivity	References
Trans-polyacetylene	Na^+^	(C_10_H_8_)Na	Solution doping	80	[56]
Poly(*p*-phenylene)	${\mathrm{AsF}}_{5}$	${\mathrm{AsF}}_{5}$	Vapor phase doping	1.5 × 10^4^	[57]
Poly(*p*-phenylene vinylene)	CH_3_SO_3_H	CH_3_SO_3_H	Non-redox doping	10.7	[58]
	${\mathrm{AsF}}_{5}$	${\mathrm{AsF}}_{5}$	Vapor phase doping	57	[59]
Poly(3-vinylperylene)	${\mathrm{Cl}}_{4}^{-}$	(C_4_H_9_)_4_N(ClO_4_)	Electrochemical doping	10^−5^	[60]
PPy	${\mathrm{AsF}}_{6}^{-},{\mathrm{PF}}_{6}^{-},{\mathrm{BF}}_{4}^{-}$	C_16_H_36_AsF_6_N, (CH_3_)_4_N(PF_6_), (C_2_H_5_)_4_N(BF_4_)	Electrochemical doping	30–100	[61]
	NSA	2-naphthalene sulfonic acid (NSA)	Electrochemical doping	1–50	[62]
	${\mathrm{Cl}}_{4}^{-}$	LiClO_4_	Electrochemical doping	65	[63]
	${\mathrm{Cl}}^{-}$	NaCl	Electrochemical doping	10	[64]
	PSS/${\mathrm{Cl}}^{-}$	PSS/FeCl_3_	Solution doping	4	[65]
	MeOH	MeOH	Vapor phase doping	0.74	[66]
	${\mathrm{HSO}}_{4}^{-}$	(C_4_H_9_)_4_N(HSO_4_)	Electrochemical doping	0.3	[61]
	C_20_H_37_O_4_SO_3_^−^	C_20_H_37_O_4_SO_3_Na	Solution doping	4.5	[67]
PANI	C_10_H_15_OSO_3_^−^	C_10_H_16_O_4_S	Solution doping	300	[68]
	HC1	HC1	Non-redox doping	10	[69]
	I_3_^−^	I_2_	Vapor phase doping	9.3	[70]
	BF_4_^−^	HBF_4_	Solution doping	(2.3 × 10^−1^)	[71]
PBTTT ^1^	FTS ^2^	C_8_H_4_F_13_SiCl_3_	Vapor phase doping	604–1.1 × 10^3^	[72,73]
Poly(2-(3-thienyloxy)ethanesulfonate)	Na_2_SO_3_	Na_2_SO_3_	Solution doping	5	[74]
PT	${\mathrm{Cl}}^{-}$	FeCl_3_	Vapor phase doping	10–25	[75]
PANI-PPy	ASPB	Anionic spherical polyelectrolyte brushes (ASPB)	Electrochemical doping	8.3	[76]

^1^ Poly(2,5-bis(3-tetradecylthiophen-2-yl)thieno[3,2-*b*]thiophene); ^2^ Tridecafluoro-(1,1,2,2-tetrahydrooctyl)-trichlorosilane.

**Table 3:** Different methods used for doping CPs.

Doping Method	Controlled Variables	Advantages	Disadvantages
Chemical doping	Vapor pressure, Exposure time to dopant	Simple way to obtain doping upon exposure of the sample to a vapor of the dopant or immersion into a solution with the dopant	Performed as slowly as possible to avoid inhomogeneous doping
			The doping levels obtained are not stable with respect to time
			Unexpected structural distortion may cause electrical conductivity decay
			Doping/de-doping shows low reversibility
Electrochemical doping	Amount of current passed	Doping level can be easily controlled by using an electrochemical cell with a controlled amount of current passed	Unexpected structural distortion may cause electrical conductivity decay
		Doping/de-doping is highly reversible and clean polymer can be retrieved	
		Can be achieved with many dopant species	
Photo doping	Radiation energy of light beam	Charge carrier is formed without chemical compound (dopants)	The electrical conductivity disappears rapidly when irradiation is discontinued due to recombination of electrons and holes
		No distortion of the material structure	
Non-redox doping	Protonic acid strength	Number of electrons generally does not change	Depends on the degree of oxidation of CPs and degree of protonation of the material
			Low conductivities are observed for some CPs
Charge-injection doping	Applying an appropriate potential on the polymer structure	Does not generate counter ions. Minimized distortion	Coulombic interaction between charge and dopant ion is very strong and can lead to change in the energetics of the system

**Table 4:** The *σ* (300K) and $\rho_{r}=\left[\rho \left(1.3K\right)/\rho \left(300K\right)\right]\;$ values for several representative CPs in the metallic, critical, and insulating regimes [77].

CP Type	Metallic		Critical		Insulating	
	$\rho_{r}$	*σ* (S·cm^−1^)	$\rho_{r}$	*σ* (S·cm^−1^)	$\rho_{r}$	*σ* (S·cm^−1^)
Polyacetylene-I_2_	<10	>5000	10–20	3–5 × 10^4^	>20	<3000
Polyacetylene-I_2_	<5	>5 × 10^4^	9.8–165	2–5 × 10^4^	>400	<2 × 10^4^
Polyacetylene-FeCl_3_	<2	>2 × 10^4^	2.6–11.4	1–2 × 10^4^	>27	<10^4^
PPV-AsF_5_	<5	300–2400	9.7–34	100–300	>50	<100
PPV-H_2_SO_4_	<2	>4 × 10^3^–10^4^	4.7–27	1000–4000	>60	<1000
PPy	<2	300–400	2–10	200–300	>10	<200
PANI	<2	250–350	2–5	200–250	>10	<200

**Table 5:** Metal or metal compound/CP nanocomposites for chemical sensors.

CPs	Metal Compounds	Target Detection	References
PANI	Pd	Methanol	[141]
PANI	Ag	Ethanol	[142]
PANI	Cu	Chloroform	[143]
PPy	ZnO	Liquefied petroleum gas	[144]
PPy	Fe_2_O_3_	CO_2_, N_2_, CH_4_, H_2_S, NH_3_ and humidity	[145,146]
PPy	Lead phthalocyanine (PbPc)	NO_2_	[147]

## Abbreviations

The following abbreviations are used in this manuscript:

CPs	Conducting polymers
PPy	Polypyrrole
PANI	Polyaniline
PT	Polythiophene
PEDOT	Poly(3,4-ethylenedioxythiophene)
PPV	Poly(*p*-phenylene vinylene)
HOMO	Highest occupied molecular orbital
LUMO	Lowest unoccupied molecular orbital
CSA	Camphor sulfonic acid
CV	Cyclic voltammetry
EDLCs	Electrochemical double layer capacitors
RGO	Graphene oxide
NCFC	Nitrogen-doped carbon fiber cloth
CNTs	Carbon nanotubes
PPCL	PPy/cellulose
FET	Field-effect transistor
CPNTs	Carboxylated polypyrrole nanotubes
GO_x_	Glucose oxidase
PSA	Prostate-specific antigen
BRCA1	Breast cancer susceptibility gene
